# Adipose-Derived Mesenchymal Stem Cells Inhibit JNK-Mediated Mitochondrial Retrograde Pathway to Alleviate Acetaminophen-Induced Liver Injury

**DOI:** 10.3390/antiox12010158

**Published:** 2023-01-09

**Authors:** Yelei Cen, Guohua Lou, Jinjin Qi, Minwei Li, Min Zheng, Yanning Liu

**Affiliations:** The State Key Laboratory for Diagnosis and Treatment of Infectious Diseases, National Clinical Research Center for Infectious Diseases, Collaborative Innovation Center for Diagnosis and Treatment of Infectious Diseases, The First Affiliated Hospital, College of Medicine, Zhejiang University, Hangzhou 310003, China

**Keywords:** mesenchymal stem cell, APAP-induced liver injury, mitochondria retrograde, ROS, DNA damage

## Abstract

Acetaminophen (APAP) is the major cause of drug-induced liver injury, with limited treatment options. APAP overdose invokes excessive oxidative stress that triggers mitochondria-to-nucleus retrograde pathways, contributing to APAP-induced liver injury (AILI). Mesenchymal stem cell therapy is a promising tool for acute liver failure. Therefore, the purpose of this study was to investigate the beneficial effects of adipose-derived mesenchymal stem cell (AMSC) therapy on AILI and reveal the potential therapeutic mechanisms. C57BL/6 mice were used as the animal model and AML12 normal murine hepatocytes as the cellular model of APAP overdose. Immunohistochemical staining, Western blotting, immunofluorescence staining, and RNA sequencing assays were used for assessing the efficacy and validating mechanisms of AMSC therapy. We found AMSC therapy effectively ameliorated AILI, while delayed AMSC injection lost its efficacy related to the c-Jun N-terminal kinase (JNK)-mediated mitochondrial retrograde pathways. We further found that AMSC therapy inhibited JNK activation and mitochondrial translocation, reducing APAP-induced mitochondrial damage. The downregulation of activated ataxia telangiectasia-mutated (ATM) and DNA damage response proteins in AMSC-treated mouse liver indicated AMSCs blocked the JNK-ATM pathway. Overall, AMSCs may be an effective treatment for AILI by inhibiting the JNK-ATM mitochondrial retrograde pathway, which improves APAP-induced mitochondrial dysfunction and liver injury.

## 1. Introduction

Acetaminophen (APAP) is a widely used analgesic and antipyretic drug that is metabolized in the liver. Because of its low cost and ease of access, APAP overdose is the most prevalent cause of acute liver failure worldwide [1]. In the liver, APAP is primarily converted to pharmacologically inactive glucuronide and sulfate conjugates, with a minor fraction being converted into a toxic intermediate, N-acetyl-p-benzoquinone imine (NAPQI), mainly by cytochrome P450 (CYP) 2E1 and CYP1A2, but toxic doses of APAP promote the production of NAPQI, leading to hepatic glutathione (GSH) depletion and protein adduct formation [2,3]. This results in mitochondrial oxidative stress and c-Jun N-terminal kinase (JNK) activation, which is a key mechanism of APAP-induced liver injury (AILI) [4]. In AILI, JNK translocation to the mitochondria is responsible for mitochondrial disruption and excessive reactive oxygen species (ROS) release [5].

The mitochondrial retrograde pathway has received increasing interests in the study of mitochondria-related diseases [6,7]. The mitochondria-to-nucleus retrograde pathway contributes to the regulation of nuclear-encoded transcription factors and coactivators that govern mitochondrial function or nuclear stress signals [8,9]. Excessive ROS, decreased ATP and mitochondrial membrane potential (ΔΨm), increased free calcium and the mitochondrial unfolded protein response all drive mitochondrial retrograde pathways [10]. ROS are key activators of mitochondrial retrograde signaling, including activating HIF-1α, Nrf2, NF-κB and the DNA damage response [11,12,13,14]. The retrograde pathway may be a novel target for treating AILI since APAP-induced mitochondrial damage possesses similar characteristics.

Mesenchymal stem cells (MSCs) are a promising tool for acute liver injury since they are easy to acquire and have the properties of self-renewable, multi-differentiation, and immunomodulation [15,16]. The beneficial effects of MSC therapy have been illustrated in liver injury by immunoregulatory function [17], secreting regenerative cytokine [18], and regulation of cell-to-cell communication by exosomes [19]. MSCs are also an ideal material for tissue engineering and reconstruction, which might be an alternative therapeutic strategy for liver transplantation [20]. For example, the combination of stem cells with 3D bioprinting is an attractive technology to obtain repeatable structures with high resolution and mimic patient-specific anatomical structures [21,22,23]. Recent studies have uncovered that MSC treatment contributes to the restoration of mitochondrial function in injured cells and has antioxidant effects on oxidative stress [24,25]. However, whether MSC therapy can affect the mitochondrial retrograde pathway in AILI is not clear. APAP overdosing can cause acute liver failure in a short period of time and the clinically approved treatment, N-acetylcysteine (NAC), has a short therapeutic window [26]. Though MSC therapy has shown promising benefits, the optimal timing of administration varies by disease [27,28,29]. Therefore, it is also important to clarify the therapeutic window of MSC treatment in AILI.

This study has investigated the effects and therapeutic window of adipose-derived MSCs (AMSCs) in AILI mice. We present evidence that the intravenous administration of AMSCs restored impaired mitochondrial function and DNA damage after APAP overdosing and identified the underlying mechanisms.

## 2. Materials and Methods

### 2.1. Animal Experiments

Male C57BL/6 mice (18–22 g, 5–7 weeks old) used in this research were obtained from GemPharmatech Co., Ltd. (Nanjing, China). The animals were housed five per cage under standard laboratory conditions, with free access to a regular commercial diet and water. The experiment was carried out in the institutional animal care and use committee of the First Affiliated Hospital of Zhejiang University. APAP (HY-66005), NAC (HY-B0215) and Anisomycin (HY-18982) were purchased from MedChemExpress (Monmouth Junction, NJ, USA). All animals were fasted overnight before APAP administration and were administered a single dose of 500 mg/kg APAP by intraperitoneal (i.p.) injection to establish a mouse model of APAP-induced liver injury. NAC (300 mg/kg via i.p. injection) and AMSCs (2 × 10^5^ via tail vein) were treated immediately after APAP administration. A JNK activator, anisomycin (20 mg/kg), was administered via i.p. injection 30 min prior to APAP injection to further investigate the role of the JNK pathway in mediating the protective effect of AMSCs. For AMSCs delayed treatment assay, AMSCs were injected 1 h, 2 h, 4 h and 6 h after APAP overdosing. Mice were killed 6 and 24 h after receiving APAP treatment. Serum samples and liver tissues were harvested. Liver tissues were flash frozen in liquid nitrogen and stored at −80 °C for further use.

### 2.2. Cell Culture and Treatment

AMSCs were obtained from inguinal adipose tissues of C57BL/6 mice, as we previously described [19]. After being digested with 0.075% collagenase type I (Sigma, St. Louis, MO, USA) and washed with PBS, cells were expanded in the murine MesenCult™ Expansion Kit (Stemcell, Vancouver, BC, Canada). AML-12 cell line was purchased from Procell Life Science & Technology Co., Ltd. (Wuhan, China) and cultured in DMEM/F12 containing 10% FBS, 10 µg/mL insulin, 5.5 µg/mL transferrin, 5 ng/mL selenium, 40 ng/mL dexamethasone and 1% penicillin-streptomycin at 37 °C in a 5% CO_2_ atmosphere. The JNK inhibitor SP600125 (HY-12041) and ATM inhibitor KU-55933 (HY-12016) were purchased from MedChemExpress (Monmouth Junction, NJ, USA). The cells were preincubated with SP600125 (10 µM) or KU-55933 (10 µM) for 1 h to inhibit JNK or ATM before being treated with 10 mM APAP or 4 µM Anisomycin for 24 h.

### 2.3. Liver Histological and Serum Biochemical Analysis

Formalin-fixed tissue samples were embedded in paraffin, and 5 μm sections were cut. Sections were stained with hematoxylin and eosin (H&E staining) for the evaluation of necrosis. Neutrophils and γH2AX positive cells were detected using a Ly6G antibody (1:400, Servicebio, Wuhan, China) and a γH2AX antibody (1:500, Huabio, Hangzhou, China) respectively. H&E staining and immunohistochemistry (IHC) analysis were evaluated by microscopy, and five visual fields were randomly selected for observation. Serum alanine aminotransferase (ALT) and aspartate aminotransferase (AST) were measured by an automatic biochemical analyzer from Rayto Life and Analytical Sciences Co., Ltd. (Shenzhen, China).

### 2.4. Transmission Electron Microscopy

The liver samples were cut into 1 mm^3^ blocks, followed by a 2 h fixation period using 2.5% glutaraldehyde/phosphate buffer. Osmium tetroxide (1%) was used for the secondary fixation. After dehydration, embedding, and staining, images of the samples were acquired using a transmission electron microscope (Hitachi HT7700, Tokyo, Japan).

### 2.5. Quantification of Reduced GSH Level in Liver

The levels of liver GSH were measured according to the instructions of the Reduced GSH Assay Kit from Nanjing Jiangcheng Bioengineering Institute (Nanjing, China). Briefly, liver tissues were homogenized in normal saline and centrifuged at 2500× *g* rpm for 10 min to a prepare 10% liver tissue homogenate. Then, 100 µL of supernatant, 100 µL of buffer, and 25 µL of chromogen were added to a well of a 96-well plate. The mixture was kept at room temperature for 5 min. Each sample was tested in triplicate and analyzed at 405 nm using the microplate reader BioTek Synergy Neo2 (Winooski, VT, USA).

### 2.6. Evaluation of Liver Tissue ATP Content

The hepatic ATP concentration was assessed with ATP Assay Kit (Beyotime Biotechnology, Shanghai, China). The liver tissue was made into 10% homogenate with lysate and centrifuged at 12,000× *g* at 4 °C for 5 min. The supernatants were quickly mixed with ATP detection working solution and detected by the luminometer Biotek Synergy Neo2. The data were normalized to the percentages of the control group.

### 2.7. Isolation of Mitochondria from Liver Tissue

Take 100 mg of fresh liver tissue and wash it with PBS once. Then mince the tissue on ice and add 1 mL pre-cooled mitochondrial isolation reagent A with PMSF from the Mitochondria Isolation Kit (Beyotime Biotechnology, Shanghai, China). Fully homogenize the tissue in an ice bath. Then, centrifuge the homogenate at 1000× *g* for 5 min at 4 °C. The supernatant was transferred to another centrifuge tube and centrifuged at 3500× *g* for 10 min. The obtained pellets were mitochondria for Western blot and JC-1 detection.

### 2.8. Mitochondrial Membrane Potential Analysis

Detection of altered ΔΨm was performed by using the JC-1 Staining Kit (Beyotime Biotechnology, Shanghai, China) according to manufacturer’s instructions. JC-1 staining of tissue was performed on isolated mitochondria by mixing 900 µL of diluted JC-1 staining working solution with 100 µL of purified mitochondria. Then, use a fluorescence spectrophotometer to measure the fluorescence intensity of JC-1 monomer and aggregate respectively. Cells were incubated with 2 mg/mL JC-1 at 37 °C for 20 min. The fluorescent images were captured using the EVOS M7000 Imaging System (Thermo Fisher, Waltham, MA, USA).

### 2.9. ROS Measurement

After thawing at room temperature, frozen slides of liver tissue were incubate in the dark at 37 °C for 30 min with dihydroethidium (DHE) staining solution (Sigma, St. Louis, MO, USA). Then they were counterstained with 4′,6-diamidino-2-phenylindole (DAPI) for 10 min at room temperature. Using fluorescent microscopy (Nikon EclipseC1, Nikon, Tokyo, Japan) to image slides. For live cells, ROS were stained by incubating with 10 µM DCFH-DA (Beyotime Biotechnology, Shanghai, China) and 2 µM mitoSOX Red (Thermo Fisher, Waltham, MA, USA) at 37 °C for 30 min. Then images were obtained by the EVOS M7000 Imaging System.

### 2.10. Western Blot Analysis

Liver tissue proteins were obtained from tissue lysates for western blotting. Protein concentration was determined using a BCA assay kit (Sigma-Aldrich, Saint Louis, MO, USA). Denatured proteins were separated on 12–20% Bis-Tris MOPS gels by SDS-PAGE and transferred to PVDF membranes. The PVDF membranes were incubated overnight at 4 °C with anti-Phospho-SAPK/JNK (Thr183/Tyr185) (1:1000, #4668, CST, Boston, MA, USA), anti-JNK (1:1000, #9252, CST, Boston, MA, USA), anti-VDAC1 (1:2000, ab154856, Abcam, Cambridge, UK), anti-Phospho-Src Family (Tyr416) (1:1000, #6943, CST, Boston, MA, USA), anti-Src (1:1000, #2123, CST, Boston, MA, USA), anti-Phospho-ATM (Ser1981) (1:400, sc-47739, Santa Cruz BioTech, Santa. Cruz, CA, USA), anti-ATM (1:4000, A1106, Sigma-Aldrich, MO, USA), anti-γH2AX (1:5000, ab81299, Abcam, Cambridge, UK), anti-H2AX (1:1000, 10856-1-AP, Proteintech, Wuhan, China), anti-p21(1:1000, ab188224, Abcam, Cambridge, UK) and anti-GAPDH (1:10,000, MB001, Bioworld Technology, Nanjing, China). The membrane-bound antibodies were detected by a hypersensitive chemiluminescence detection reagent from Fude Biological Technology Co., Ltd. (Hangzhou, China).

### 2.11. Immunocytochemistry

AML12 cells were fixed with fresh 4% paraformaldehyde for 10 min, and were permeabilized with 0.1% Triton X-100 in PBS for 10 min. After blocking with 1% BSA and 22.52 mg/mL glycine in PBS for 1 h, anti-Phospho-ATM (Ser1981) (1:50, sc-47739, Santa Cruz BioTech, Santa. Cruz, CA, USA) and anti-γH2AX (1:100, ab81299, Abcam, Cambridge, UK) were added and incubated overnight at 4 °C. The following day, plates were washed three times with PBS. After rinsing, secondary antibodies were applied for 1 h at room temperature, followed by three additional washes with PBS. Nuclei were counterstained with DAPI. Images were acquired by the EVOS M7000 Image System with a 40× objective.

### 2.12. RNA Sequencing and Analysis

Total RNA was extracted, and the RNA integrity was measured using the Agilent 2100 bioanalyzer system (Santa Clara, CA, USA). Libraries of amplified RNA for each of the nine samples were prepared in accordance with the Illumina protocol. The cDNA library construction and sequencing were conducted based on the procedures by Novogene Co., Ltd. (Beijing, China). Differential expression analysis of two groups (three biological replicates per group) was performed using the DESeq2 R package (1.20.0). Genes with padj ≤ 0.05 and |log2 (foldchange)| ≥ 1 were considered significantly differential expressed genes (DEGs). The database annotation of the pathways and functions of DEGs was carried out by Kyoto Encyclopedia of Genes and Genomes (KEGG) analysis and reactome pathway enrichment analysis. Gene set enrichment analysis (GSEA) is a computational method for determining if a predefined gene set can show a statistically significant difference between two biological states, which can include subtle expression changes. In this study, the local version of the GSEA analysis tool (http://www.broadinstitute.org/gsea/index.jsp, accessed on 22 June 2022) and predefined gene sets of mitochondrial pathways in MitoCarta 3.0 (https://www.broadinstitute.org/mitocarta/mitocarta30-inventory-mammalian-mitochondrial-proteins-and-pathways, accessed on 22 June 2022) were used. Heat maps and volcano plots were generated using TBtools (version 1.098774) [30].

### 2.13. Statistical Analysis

The data are expressed as means ± SD. Student’s *t* tests and one-way ANOVA followed by Tukey’s test were used to compare the differences between each group. GraphPad Prism 8.0 (GraphPad Software, La Jolla, CA, USA) was used for statistical analysis.

## 3. Results

### 3.1. AMSC Injection Effectively Ameliorated APAP Induced Liver Injury

**Figure 1:**
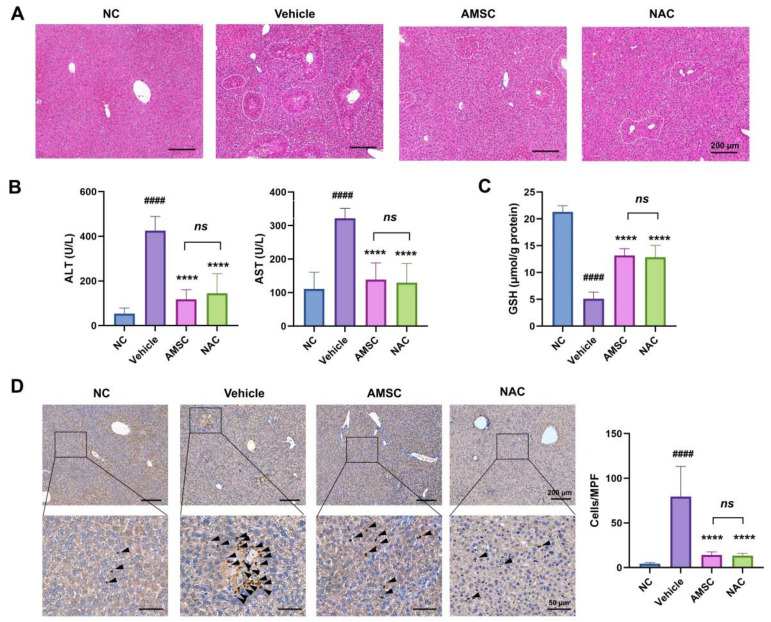
Effects of AMSC therapy on mice exposed to 500 mg/kg APAP. (**A**) Representative HE-staining and quantitation of the necrosis area in liver sections obtained from normal control (NC group), APAP-overdosed (vehicle group), AMSC-injected (AMSC group), and NAC-treated (NAC group) mice for 24 h. (**B**) Serum ALT and AST. (**C**) Hepatic GSH level. (**D**) Representative Ly6G IHC analysis. The positive cells were pointed out by arrowheads. Quantification of positive cells in each group per medium power field (MPF) using a 20× objective. Data were expressed as mean ± SD; n = 5; *#### p* < 0.0001 vs. NC group; **** p < 0.0001 versus vehicle group; ns: no significance versus NAC group.

To evaluate the effect of AMSC therapy on APAP-induced liver injury, we collected liver tissues and serum samples from APAP-overdosed, AMSC-treated mice and NAC-treated mice. The H&E sections showed that the intense centrilobular necrosis was much more reduced in AMSC-treated mice after a 24 h APAP challenge (Figure 1A). Similarly, serum AST and ALT levels, two indicators of liver damage, were drastically decreased after 24 h of AMSC treatment (Figure 1B). Then we analyzed the reduction in antioxidant GSH level after 6 h of APAP overdose, which is a critical step in APAP-induced liver injury. The total GSH levels in the liver revealed that AMSCs partially rescued the GSH depletion (Figure 1C). The number of neutrophils infiltrating as indicated by Ly6G staining was decreased in AMSC-treated mice, suggesting a significant reduction in liver inflammation (Figure 1D). Moreover, these therapeutic effects of AMSC therapy are comparable to those of NAC in clinical use. These results suggested that AMSCs are an effective therapy for AILI.

### 3.2. AMSCs Rescue Mitochondrial Function during APAP-Induced Liver Injury

**Figure 2:**
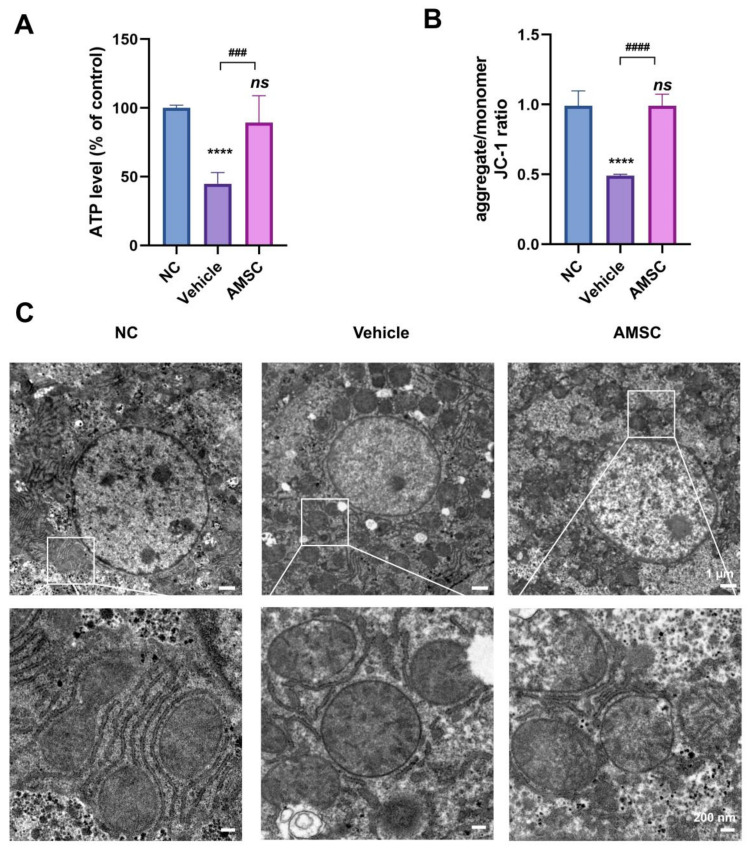
AMSC treatment prevents APAP-induced mitochondrial dysfunction. (**A**) Hepatic ATP levels in each group. Mice were sacrificed after APAP overdosing and AMSC treatment for 6 h. (**B**) JC-1 staining for detecting ΔΨm in each group. (**C**) Distinctive mitochondrial morphological changes were detected by electron microscopy in mouse livers treated with APAP and AMSC. Data are expressed as mean ±SD; n = 5; **** p < 0.0001 and ns: no significance vs. NC group; *### p* < 0.001 and *#### p* < 0.0001 vs. vehicle group. NC group: normal control mice; vehicle group: APAP-overdosed mice; AMSC group: AMSC-treated mice.

We next tested whether AMSC treatment could ameliorate injured mitochondrial function triggered by APAP overdosing. Mice were injected with a single dose of 500 mg/kg APAP to induce acute liver injury, and some of them were treated with AMSCs. After 6 h, the mice were sacrificed, and liver tissues were collected to test mitochondrial activity. The elevated ATP level was observed after AMSC treatment which suggested a protective effect on ATP synthesis in mitochondria (Figure 2A). We also estimated the effects of AMSCs on mitochondrial membrane potential (ΔΨm) by staining isolated mitochondria from liver tissue with JC-1. AMSC treatment can alleviate APAP-induced loss of hepatic ΔΨm, which is essential for the oxidative phosphorylation process (Figure 2B). Results from electron microscopy investigations further revealed the changes in mitochondrial morphology. We observed swollen mitochondria with blunted cristae in the APAP group, while the ultrastructures of mitochondria in the AMSC group were quite similar to those in the NC group (Figure 2C). Together, treating with AMSCs had a protective effect against APAP-induced mitochondrial injury.

### 3.3. The Therapeutic Window of AMSC Therapy Is Related to the Mitochondrial Retrograde Pathway

**Figure 3:**
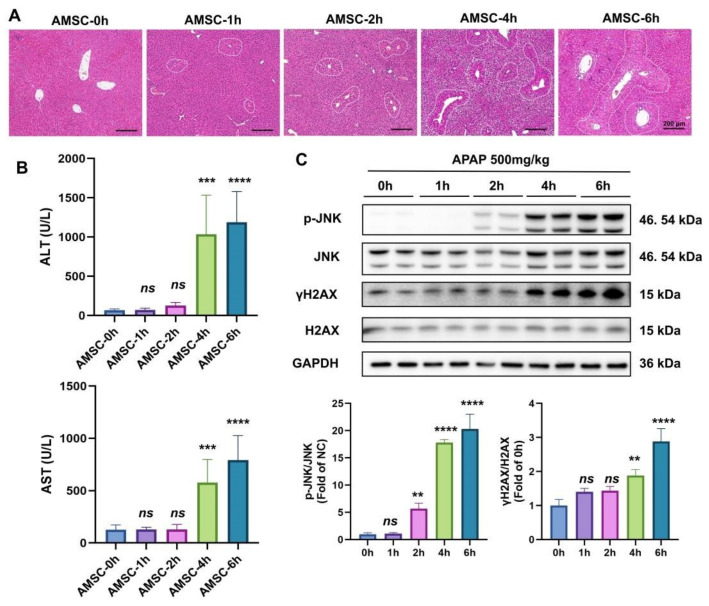
The APAP-induced mitochondrial retrograde pathway limits therapeutic potential of AMSCs. (**A**) Representative HE-staining of delayed AMSCs injection at 1 h, 2 h, 4 h, and 6 h after APAP exposure. (**B**) Serum ALT and AST. (**C**) The protein expression levels of p-JNK and γH2AX at different times after the APAP challenge. Data are presented as the mean ± SD; n = 3–5; ** p < 0.01, *** p < 0.001, **** p < 0.0001 and ns: no significance vs. 0 h group.

To explore the therapeutic window of AMSCs administration, we performed delayed AMSC treatment at different time points after APAP overdosing. After 24 h of APAP intoxication, liver histology showed that 1 h and 2 h of delayed administration significantly reduced liver necrosis, but massive necrosis was observed in the 4 h and 6 h delayed treatment groups (Figure 3A). Consistent with the histological analysis, serum AST and ALT levels were significantly elevated in the 4 h and 6 h treated groups. These results indicated that AMSC treatment would be ineffective 4 h after APAP overdosing (Figure 3B). Previous studies have shown JNK activation is key to mitochondrial damage caused by APAP toxicity [5,31]. The mitochondrial retrograde pathway initiated by JNK results in the formation of cytoplasmic chromatin fragments and the induction of DNA damage, which is responsible for APAP-induced hepatocyte injury and regenerative failure [32,33]. Figure 3C suggested that the expression of p-JNK gradually increased from 0 h to 2 h and was sharply activated at 4 h. γH2AX, a marker of cytoplasmic chromatin fragments formation and DNA damage, showed the same trend as the level of p-JNK, which was abundantly expressed 4 to 6 h after APAP poisoning. This suggested that JNK was massively activated at the time point 4 h after the APAP challenge and triggered a mitochondria-to-nucleus retrograde signaling pathway. Taken together, the limited therapeutic window of AMSC therapy may be related to the JNK-mediated mitochondria-to-nucleus retrograde pathway.

### 3.4. AMSC Treatment Reduces JNK Activation and Mitochondrial Translocation

**Figure 4:**
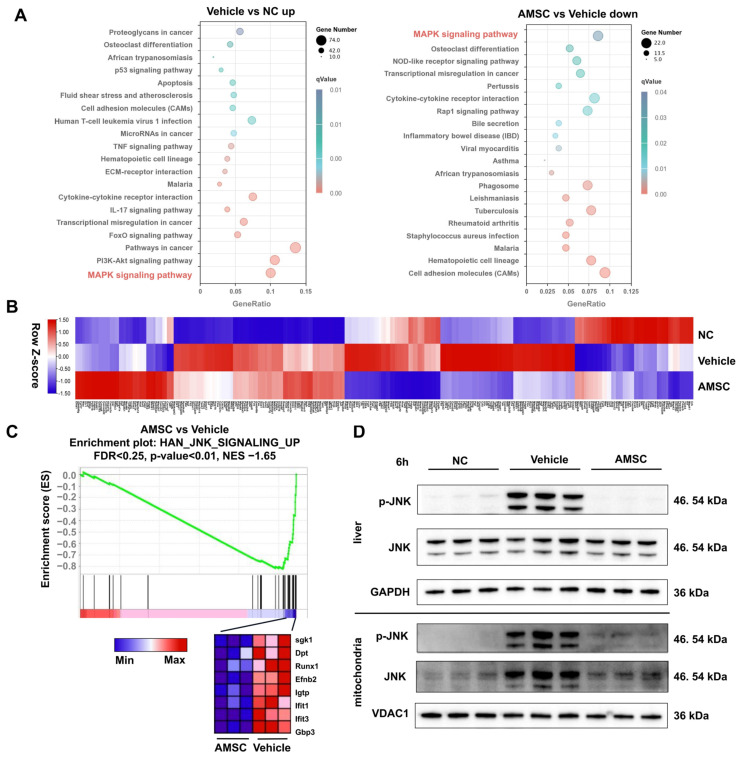
AMSCs inhibit JNK activation and mitochondrial translocation in AILI. (**A**) The enrichment maps of KEGG pathways of differentially expressed genes in the vehicle group versus the NC group and the AMSC group versus the vehicle group. (**B**) Heat maps of the MAPK signaling pathway gene subset in each group plotted with cohort means of Z scores. (**C**) The pathway of JNK signaling up was among significant differences in GSEA of AMSC group vs. vehicle group. Genes with the highest enrichment score are shown as a gene expression-based heat map. (**D**) The Western blot analysis of p-JNK level in isolated mitochondria and liver tissue after 6 h APAP exposure and AMSC treatment. NC group: normal control mice; vehicle group: APAP-overdosed mice; AMSC group: AMSC-treated mice.

To further clarify the potential mechanisms of AMSC therapy in AILI, we performed RNA-seq analysis using samples after 6 h APAP overdosing and AMSC treatment. The MAPK pathway was significantly enriched by KEGG enrichment analysis of the downregulated differential genes after AMSC treatment. Similarly, KEGG enrichment of the upregulated differential genes in the APAP group compared with the NC group also showed a significant enrichment in the MAPK pathway (Figure 4A). The heat map of the MAPK pathway showed some abnormally activated genes caused by APAP toxicity were restored by AMSC treatment (Figure 4B). These results indicated that AMSC treatment attenuated the MAPK pathway triggered by APAP hepatotoxicity. JNK is a subtype of the MAPK signaling pathway and plays a key role in APAP-induced mitochondrial defects. Further analysis using GSEA showed that the JNK upregulation pathway gene set was significantly reduced after AMSC treatment (Figure 4C). Given that JNK mitochondrial translocation plays a central role in APAP-induced disturbed mitochondrial function and cell death, we sought validation by performing Western blotting on liver tissue and isolated mitochondria. Results showed that p-JNK expression in the liver and its translocation to mitochondria were both significantly reduced after AMSC treatment (Figure 4D). Therefore, AMSC treatment inhibited activation and mitochondrial translocation of JNK in the liver tissues of the AILI mice model.

### 3.5. AMSC Treatment Inhibits JNK-Mediated ROS Generation and Restores Mitochondrial Activity

**Figure 5:**
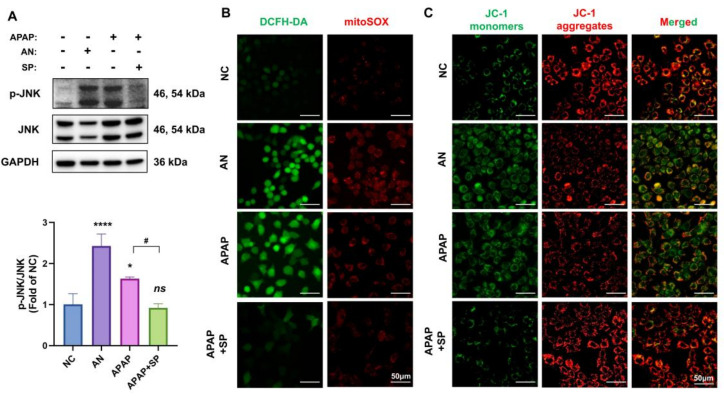
Inhibition of JNK activation alleviates APAP-induced mitochondrial dysfunction in AML12 cells. (**A**) Western blot analysis of p-JNK and JNK levels in AML12 cells treated with anisomycin, APAP, and SP600125 for 24 h. (**B**) The cells were stained by DCFH-DA and MitoSOX to visualize intracellular ROS. (**C**) The representative fluorescent microscopic analysis of ΔΨm by JC-1 staining of each group. Data are presented as the mean ± SD; n = 3; * p < 0.05, **** p < 0.0001 and ns: no significance vs. NC group; *# p* < 0.05 vs. APAP group. AN: anisomycin; SP: SP600125.

We first examined in AML12 cells (an immortal hepatocyte cell line), that JNK can be activated by APAP stimulation, such as the JNK agonist (anisomycin), while APAP-induced JNK activation was inhibited after pretreatment with the JNK antagonist (SP600125), which was similar to the in vivo results of AMSC therapy we verified above (Figure 5A). Then we measured the overall cellular level of ROS and mitochondrial-derived ROS using DCFH-DA and mitoSOX in AML12 cells treated with anisomycin, APAP, and SP600125 for 24 h. Fluorescence images showed a great increase of intracellular and mitochondrial ROS content in both cells treated with anisomycin and APAP, while SP600125 effectively reduced the ROS accumulation in APAP-injured cells (Figure 5B). Cells were stained with JC-1 for detecting ΔΨm, a global indicator of mitochondrial function. JC-1 monomers increased but JC-1 aggregates decreased after treating with anisomycin and APAP, which suggested a significant reduction in ΔΨm. However, pretreatment with SP600125 can restore the diminished ΔΨm caused by APAP overdose (Figure 5C). Together, these results demonstrated that APAP-induced ROS production and mitochondrial dysfunction could be prevented by inactivating JNK.

**Figure 6:**
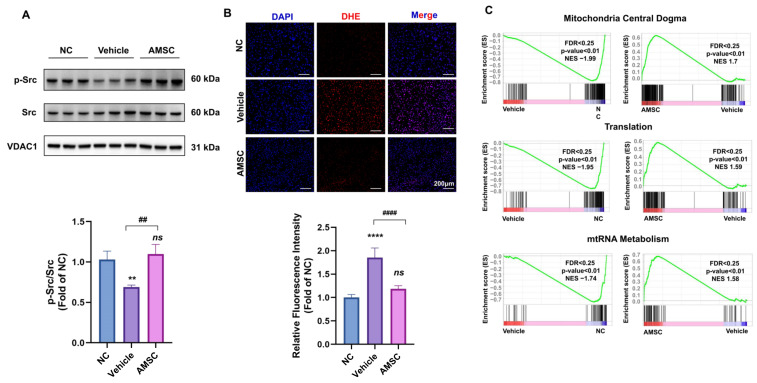
AMSCs inhibit ROS production and restore mitochondrial function. (**A**) Western blot analysis of p-Src and Src levels in isolated mitochondria from mouse liver tissues after treating with APAP and AMSCs for 6 h. (**B**) The measurement of ROS content in liver tissue by DHE staining and quantification by measuring the mean gray value of DHE. (**C**) GSEA plots of significantly different mitochondrial pathway gene sets in the vehicle group versus the NC group and the AMSC group versus the vehicle group. Data are presented as the mean ± SD; n = 3–5; ** p < 0.01, **** p < 0.0001 and ns: no significance vs. NC group; *## p* < 0.01 and *#### p* < 0.0001 vs. APAP group. NC group: normal control mice; vehicle group: APAP-overdosed mice; AMSC group: AMSC-treated mice.

Next, we investigated whether AMSC treatment could alleviate the JNK-mediated ROS activation and mitochondrial dysfunction in AILI mice. It has been reported that p-JNK translocation to the outer mitochondrial membrane causes Src inactivation in mitochondria, which accounts for increased ROS production and impaired mitochondrial function [34]. Western blot analysis of liver tissues showed that AMSC treatment effectively alleviated the inactivation of Src in the mitochondria caused by APAP overdose (Figure 6A). Excessive APAP, which caused massive ROS production in liver tissues, was also alleviated after AMSC treatment (Figure 6B). Then, we performed GSEA on mitochondrial pathways collected in the MitoCarta 3.0 inventory [35]. AMSC treatment appeared to have a beneficial role in mitochondrial activity, as we found augmented gene expression associated with mitochondrial central dogma, translation, and mtRNA metabolism in AMSC-treated mice (Figure 6C). Therefore, AMSC therapy prevented JNK-mediated mitochondrial damage, which played a key role in triggering the mitochondrial retrograde pathway of APAP-induced mouse hepatotoxicity.

### 3.6. AMSC Treatment Blocks the JNK-ATM Mitochondrial Retrograde Pathway

**Figure 7:**
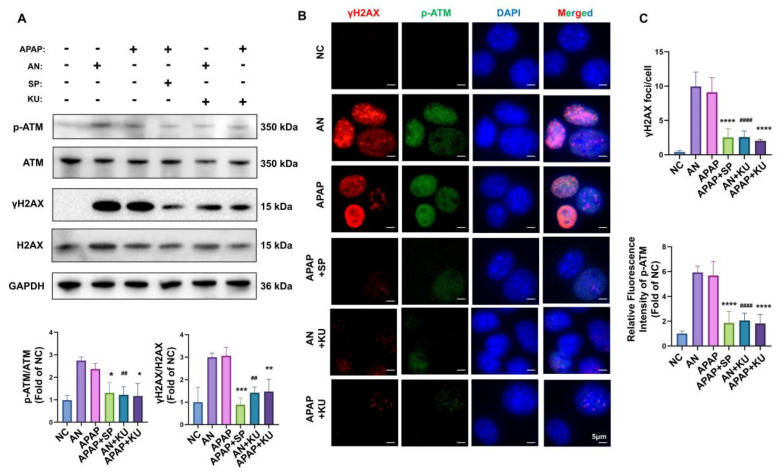
APAP-induced ATM retrograde pathway can be inhibited by inactivating JNK in AML12 cells. (**A**) Immunoblots and quantitative histograms showing the results of Western blotting analysis for the expression of p-ATM, ATM, γH2AX, and H2AX proteins. (**B**) Representative images showing the immunofluorescence staining for γH2AX (red) and p-ATM (green) among different groups. (**C**) The number of γH2AX foci per nucleus was quantitated using ImageJ (Fiji). In each sample, at least 100 nuclei cells were scored. The expression of p-ATM was quantified by measuring the mean gray value. Data are presented as the mean ± SD; n = 3; * p < 0.05, ** p < 0.01, *** p < 0.001 and **** p < 0.0001 vs. APAP group; *## p* < 0.01 and *#### p* < 0.0001 vs. AN group. AN: anisomycin; SP: SP600125; KU: KU-55933.

JNK activation and sustained ROS have been shown to drive the mitochondrial retrograde pathway, which invokes the ataxia telangiectasia mutated (ATM)-induced DNA damage response and triggers cell damage [8,14,32]. As we tested above, γH2AX (a substrate of ATM) and p-JNK showed the same trend in the course of AILI. To determine how JNK mediates the mitochondrial retrograde pathway in APAP-induced hepatotoxicity, we treated anisomycin-treated and APAP-injured AML12 cells with SP600125 and an ATM inhibitor, KU-55933. As expected, the expressions of p-ATM and γH2AX were significantly increased in AML12 cells after treating with APAP which was consistent with anisomycin stimulation. SP600125 markedly inhibited this increase in APAP-injured cells, which suggested that the ATM retrograde pathway caused by APAP is through JNK activation. Moreover, KU-55933 significantly downregulated the γH2AX expression induced by APAP and anisomycin (Figure 7A). Immunofluorescence images also showed that γH2AX formed distinct foci and p-ATM expression was elevated in the nucleus after APAP injury. These were abrogated by SP600125 and KU-55933 (Figure 7B,C). Taken together, the immunofluorescence and Western blot analysis data support the idea that activated JNK triggers mitochondria retrograde pathway via the ATM.

**Figure 8:**
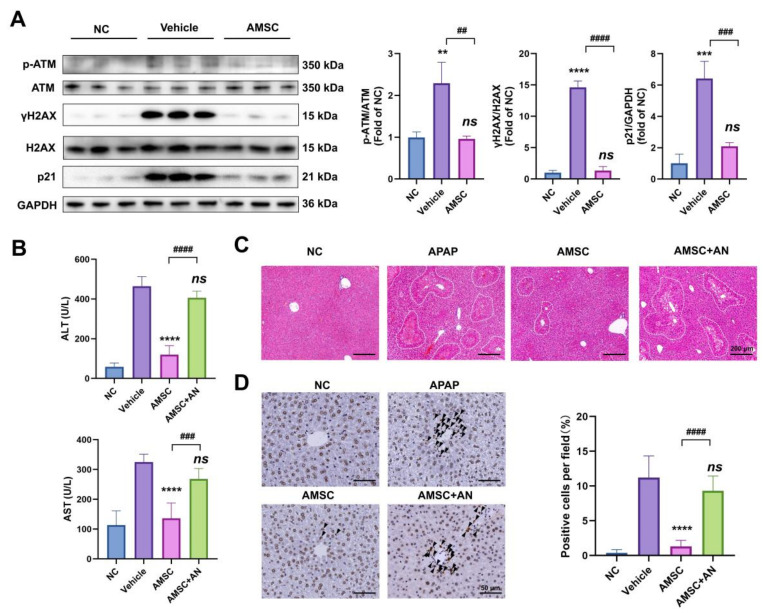
AMSCs inhibit the JNK-ATM mitochondrial retrograde pathway in AILI. (**A**) Immunoblots and quantitative histograms showing the results of Western blotting analysis for p-ATM, ATM, γH2AX, H2AX and p21 protein expression. (**B**) The AST and ALT levels after treating with AMSCs and AN. (**C**) Representative H&E staining of each group. (**D**) Liver tissues were stained for γH2AX and quantified. Representative IHC images are shown. Data are presented as the mean ± SD; n = 3–5; ** p < 0.01, *** p < 0.001, **** p < 0.0001 and ns: no significance vs. NC group and vehicle group; *## p* < 0.01, *### p* < 0.001 and *#### p* < 0.0001 vs. APAP group and AMSC + AN group. AN: anisomycin; NC group: normal control mice; vehicle group: APAP-overdosed mice; AMSC group: AMSC-treated mice; AMSC + AN group: AMSC-injected mice pretreated with anisomycin.

Then we found that AMSC treatment significantly downregulated the expression of p-ATM, since AMSC therapy inhibited sustained JNK activation, as we verified above. The reduction of γH2AX and p21 further illustrated that AMSCs markedly reduced APAP-induced DNA damage and greatly reduced hepatocyte necrosis (Figure 8A). Next, we pretreated mice receiving AMSC therapy with anisomycin, a JNK agonist. The AN group exhibited elevated ALT and AST (Figure 8B). Similar results were also revealed in histological analysis, for we detected massive liver necrosis after reactivating JNK in AMSC treatment compared with the AMSC and NC groups (Figure 8C). The γH2AX-positive cells were strongly suppressed by AMSC treatment but reappeared after restoring JNK activation (Figure 8D). Therefore, AMSC treatment effectively inhibited the JNK-ATM mitochondrial retrograde pathway as well as prevented JNK-mediated ROS production and mitochondrial damage, thereby rescuing the acute liver injury induced by APAP.

## 4. Discussion

APAP overdosing causes mitochondrial dysfunction and still lacks effective treatments [36]. Recently, therapies targeting mitochondrial damage and retrograde pathways have emerged in disease treatment studies [6,37]. In this study, we showed that AMSC therapy may serve as an effective treatment for AILI through inhibiting the JNK-ATM mitochondrial retrograde pathway and clarified that this is related to its limited therapeutic window.

MSC therapy targeting drug-induced liver injury involves multiple mechanisms, such as promoting liver regeneration and its anti-inflammatory capacity [38]. We found that AMSCs injection prevented reductions in ATP and ΔΨm and restored mitochondrial morphology. Thus, these results revealed a new mechanism of AMSCs in AILI by restoring mitochondrial function. However, the therapeutic effects of AMSC therapy were restricted to the first 4 h of the APAP injury. This may be related to APAP-induced JNK activation, which dominates the collapse of mitochondrial function and cell death in APAP overdosing [37]. As we expected, AMSCs injection inhibited JNK activation and its translocation to the mitochondria in the context of APAP overdose. Specifically, JNK interacts with an outer mitochondrial membrane protein and inactivates Src in mitochondria to inhibit electron transport and increase ROS release [34]. This has also been verified in our study, which showed that AMSCs restored inactivated Src and ROS accumulation. The GSEA of RNA-seq on APAP-overdosed and AMSC-treated mouse liver samples also indicated the beneficial role of AMSC therapy in APAP-induced mitochondrial dysfunction via inhibiting JNK.

Recent evidence has highlighted the mitochondrial retrograde pathway mediated by JNK may be responsible for APAP-induced DNA damage and impaired liver regeneration [33]. In AILI, the JNK-mediated mitochondrial retrograde signaling pathway drives cytoplasmic chromatin fragments formation and the expression of the DNA damage markers γH2AX and p21 [32]. According to our findings, the rising trend of γH2AX in AILI is consist with JNK activation. The in vitro experiment showed that blocking JNK in APAP-injured hepatocytes could abolish the increased expression of γH2AX. ATM is a sensor for oxidative stress that can be activated by excessive ROS and dysfunctional mitochondria [39,40]. It has been reported that ATM was activated by APAP exposure and increased the expression of its substrate, γH2AX [41]. A recent study has shown JNK signaling is essential for ATM-mediated nuclear and cytoplasmic stress signals [14]. We tested APAP-injured hepatocytes with the JNK inhibitor and the ATM inhibitor to verify that APAP induced a DNA damage response through the JNK-ATM retrograde pathway. Downregulation in p-ATM, γH2AX, and p21 expression in liver tissue of APAP overdosed mice elucidated the protective role of AMSCs by blocking the JNK-ATM retrograde pathway, which was further tested by treating AMSC-injected mice with the JNK agonist.

Although AMSCs transplantation in the early stages of APAP liver injury had significant efficacy, this was lost after 4 h of AILI. In our study, the JNK mitochondrial translocation and JNK-mediated mitochondrial retrograde pathway may account for the limited therapeutic time window of AMSC treatment. Since MSCs modifications by genetic editing and preconditioning are promising methods of improving MSC therapy efficacy in challenging conditions [42], it may be a possible way to broaden the therapeutic window for AILI, such as by enhancing the ability to target the mitochondrial retrograde pathway-related molecules. Despite the fact that we revealed that the AMSC treatment inhibited the JNK-mediated ATM retrograde pathway of APAP toxicity, the mitochondrial retrograde pathways in AILI may not be limited to this one. For example, APAP overdosing stabilizes and promotes nuclear factor erythroid 2-related factor 2 translocation to the nucleus, triggering a retrograde pathway [43,44]. So AMSC therapy might exert effects on AILI through other retrograde pathways.

The JNK activation in AILI is mainly attributed to the metabolism of APAP to the toxicant NAPQI by CYP2E1 [5]. Thus, we performed reactome pathway enrichment analysis of DEGs from the AMSC-treated group versus the APAP-overdosed group, and found that cytochrome P450-arranged by substrate type and CYP2E1 reactions were among the most significantly enriched pathways after 6 h of APAP challenge (*p* < 0.05) (Figure S1A). The heat map of cytochrome P450-arranged by substrate type pathway and volcano plot of DEGs also showed that AMSC therapy increased the expression of CYP2E1 mRNA (Figure S1B,C). Though the protein expression of CYP2E1 was not changed 6 h after APAP exposure, APAP overdose led to a significant reduction in hepatic CYP2E1 after 24 h (Figure S1D). This was consistent with previous studies and might be due to the serious hepatocellular injury and changes of hepatic endoplasmic reticulum [45,46,47,48]. AMSCs rescued the change of CYP2E1 by APAP overdose, which suggested that AMSC therapy may inhibit JNK activation by affecting the CYP genes involved in APAP conversion to NAPQI. The interactions between accumulated ROS and JNK contribute to sustaining JNK activation [34]. Several studies have shown that MSC therapy could increase antioxidants such as superoxide dismutase [49], catalase [50] and GSH [51]. We also found AMSC injection could restore the GSH in AILI (Figure 1C). Thus, AMSC treatment may inhibit JNK activation through multifaceted effects.

In summary, we have presented in vitro and in vivo data demonstrating that AMSC therapy could be a potential therapeutic for early-stage AILI through inhibiting the JNK-ATM pathway, which is related to restoring mitochondrial disability and reducing DNA damage response through the mitochondrial retrograde pathway. These findings promote the understanding of the underlying mechanisms of MSC treatment for acute liver failure.

## Data Availability

The RNA-Seq data discussed in this publication have been deposited in NCBI’s Gene Expression Omnibus [52] and are accessible through GEO Series accession number GSE218879 (https://www.ncbi.nlm.nih.gov/geo/query/acc.cgi?acc=GSE218879, accessed on 13 December 2022).

## Supplementary Materials

The following are available online at https://www.mdpi.com/article/10.3390/antiox12010158/s1. Figure S1. AMSC therapy may affect the metabolism of APAP to inhibit JNK activation. (A) Reactome pathway enrichment analysis of the upregulated DEGs of AMSC group versus vehicle group. (B) The heat map of cytochrome P450-arranged by substrate type pathway of each group. (C) The volcano plot of DEGs highlighting significant genes from cytochrome P450-arranged by substrate type pathway. (D) The expression of hepatic CYP2E1 after treating with 500 mg/kg APAP for 6 h and 24 h in each group. Data are presented as the mean ± SD; n = 3; *** p* < 0.01 and ns: no significance vs. NC group; *#p* < 0.05 and ns: no significance vs. APAP group. NC group: normal control mice; vehicle group: APAP-overdosed mice; AMSC group: AMSC-treated mice.
