# Supplementary material for: Adipose-Derived Mesenchymal Stem Cells Inhibit JNK-Mediated Mitochondrial Retrograde Pathway to Alleviate Acetaminophen-Induced Liver Injury
Source: Antioxidants (Basel). 2023 Jan 9;12(1):158. doi: 10.3390/antiox12010158 (PMC9854665; doi:10.3390/antiox12010158)
Supplement: Supplementary file 1 [file antioxidants-2112681-supplementary.pdf › antioxidants-2112681-supplementary.pdf]

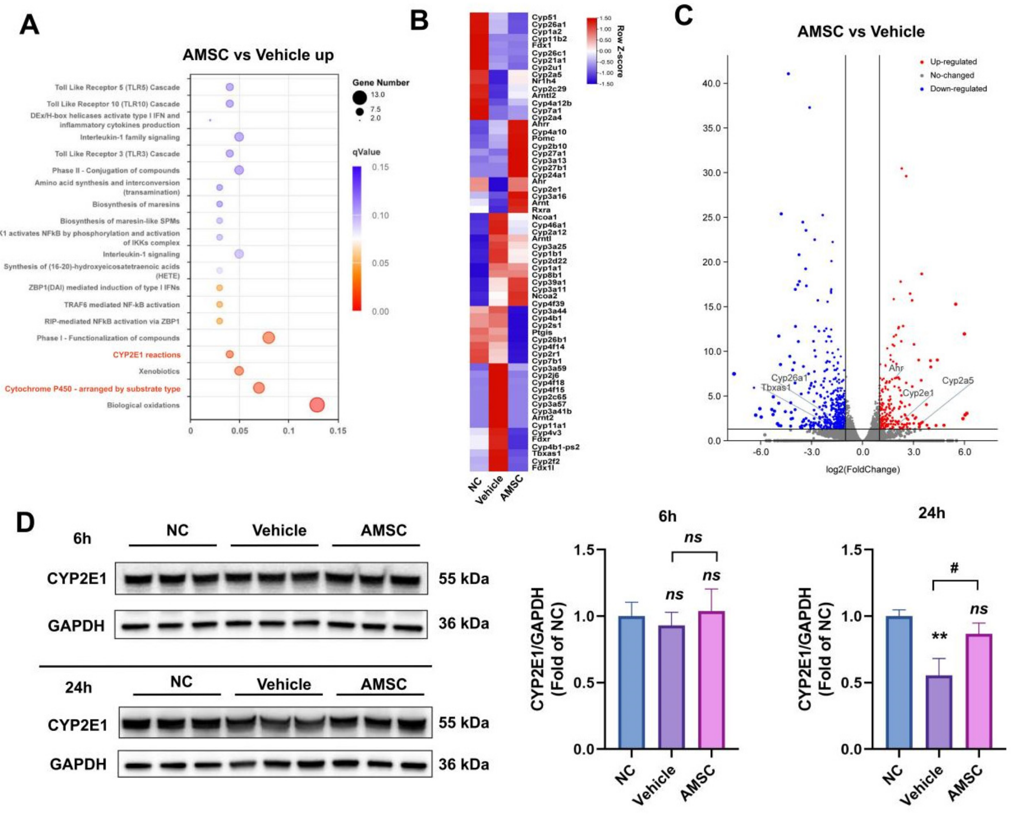

Figure S1. AMSC therapy may affect the metablism of APAP to inhibit JNK activation. (A) Reactome pathway enrichment analysis of the upregulated DEGs of AMSC group versus vehicle group. (B) The heat map of cytochrome P450 - arranged by substrate type pathway of each group. (C) The volcano plot of DEGs highlighting significant genes from cytochrome P450 - arranged by substrate type pathway. (D) The expression of hepatic CYP2E1 after treating with 500mg/kg APAP for 6 h and 24 h in each group. Data are presented as the mean ± SD; n = 3; $**p < 0.01$ and ns: no significance vs. NC group; $\#p < 0.05$ and ns vs. APAP group. NC group: normal control mice; Vehicle group: APAP-overdosed mice; AMSC group: AMSCs-treated mice.
